# Current tobacco use and SARS-CoV-2 infection in two Norwegian population-based cohorts

**DOI:** 10.1186/s12889-023-15822-5

**Published:** 2023-05-10

**Authors:** Ida Henriette Caspersen, Lill Trogstad, Maria Rosaria Galanti, Sakari Karvonen, Sebastián Peña, Ahmed Nabil Shaaban, Siri E. Håberg, Per Magnus

**Affiliations:** 1 Centre for Fertility and Health, Norwegian Institute of Public Health, Postbox 222 Skøyen, N-0213 Oslo, Norway; 2 Department of Infectious Disease Epidemiology and Modelling, Norwegian Institute of Public Health, Postbox 222 Skøyen, N-0213 Oslo, Norway; 3 Department of Global Public Health, Karolinska Institutet, SE-171 77 Stockholm, Sweden; 4 Centre for Epidemiology and Community Medicine, Stockholm Region, Solnavägen 1E (Torsplan), 113 65 Stockholm, Sweden; 5 Department of Public Health and Welfare, Finnish Institute for Health and Welfare, Postbox 30, 00271 Helsinki, Finland

**Keywords:** Smoking, snuff, snus, covid-19, MoBa, The Norwegian Mother, Father and Child Cohort Study, NorFlu

## Abstract

**Background:**

Clear evidence of an increased risk for SARS-CoV-2 infection among smokers has not been established. We aimed to investigate associations between cigarette smoking or use of snus (snuff) and other nicotine-containing products and a positive SARS-CoV-2 test, taking test behavior into account.

**Methods:**

Current tobacco use and testing behavior during the pandemic were recorded by adult participants from the Norwegian Mother, Father and Child Cohort Study and The Norwegian Influenza Pregnancy Cohort. SARS-CoV-2 infection status was obtained from The Norwegian Surveillance System for Communicable Diseases (MSIS) in May 2021 (*n* = 78,860) and antibody measurements (*n* = 5581). We used logistic regression models stratified by gender and adjusted for age, education, region, number of household members, and work situation.

**Results:**

Snus use was more common among men (26%) than women (9%) and more prevalent than cigarette smoking. We found no clear associations between cigarette smoking or snus and a COVID-19 diagnosis among men. Associations among women were conflicting, indicating that cigarette smoke was negatively associated with a diagnosis (OR 0.51, 95% CI 0.35, 0.75), while no association was found for snus use (OR 1.07, 95% CI 0.86, 1.34). Compared with non-users of tobacco, both cigarette smokers and snus users had increased odds of being tested for SARS-CoV-2.

**Conclusions:**

Cigarette smoking, but not snus use, was negatively associated with SARS-CoV-2 infection in women. The lack of an association between snus use and SARS-CoV-2 infection in this population with prevalent snus use does not support the hypothesis of a protective effect of nicotine.

**Supplementary Information:**

The online version contains supplementary material available at 10.1186/s12889-023-15822-5.

## Background

The novel coronavirus disease 2019 (COVID-19), induced by severe acute respiratory syndrome coronavirus 2 (SARS-CoV-2) was declared a pandemic by the World Health Organization on 11^th^ March 2020. By December 2022, WHO have reported more than 640 million cases and six million deaths globally [1].

Because tobacco smoking is an important risk factor for respiratory disorders [2], concern has been raised that smokers are at increased risk of SARS-CoV-2 infection, severity of COVID-19 disease and mortality [3–5]. While several studies have shown associations between tobacco smoking and poorer disease prognoses in COVID-19 patients, clear evidence of a higher risk of acquiring the infection among smokers has not been established [6].

Snus (snuff) is a moist smokeless tobacco extensively used by Norwegian adults with around 13% daily users in 2020, and more commonly used among men (19%) than women (7%) [7]. Nowadays, snus use is even more common than smoking as about 9% of Norwegians smoke daily [7]. Snus provides an equivalent or higher absorbance of nicotine compared to cigarettes [8], but without the toxic combustion yields from smoking. Therefore, snus use is a more refined proxy of nicotine exposure which may expand the understanding of the role of tobacco use in relation to COVID-19.

The aim of this study was to investigate associations between cigarette smoking or use of other nicotine-containing products (including snus) and a positive SARS-CoV-2 test during March 2020 to May 2021, as measured by i) a COVID-19 diagnosis obtained by linkage to The Norwegian Surveillance System for Communicable Diseases (MSIS) and ii) presence of SARS-CoV-2 antibodies in serum. A secondary aim was to examine associations between tobacco use and having been tested for SARS-CoV-2 in the same period, to explore if the risk of getting a registered diagnosis could be explained by testing behavior. We examine these associations cross-sectionally among participants from two large pre-existing cohorts, at a time point covering the pre- and early vaccination periods of the pandemic in Norway.

## Methods

### Study design

The current study was based on The Norwegian Mother, Father and Child Cohort Study (MoBa) [9] and The Norwegian Influenza Pregnancy Cohort (NorFlu) [10], with linkages to The Norwegian Surveillance System for Communicable Diseases (MSIS), The Norwegian Immunisation Registry (SYSVAK), The Medical Birth Registry of Norway, and The Norwegian Population Registry. MoBa is an ongoing population-based pregnancy cohort study conducted by the Norwegian Institute of Public Health. Participants were recruited from all over Norway from 1999–2008 [11]. The cohort now includes 114,500 children, 95,200 mothers and 75,200 fathers. NorFlu was initiated during the swine flu pandemic (A H1N1(pdm09)) in 2009/2010, and pregnant women were invited to participate [12]. NorFlu was established to examine associations between influenza and influenza vaccinations during pregnancy and a series of outcomes. About 4500 mother–child pairs are included. In both MoBa and Norflu, the women consented to participation in 41% of the pregnancies. Both cohorts are followed by questionnaires, registry linkages and invitations into different sub-studies.

### Study population

Sub-studies were initiated in both cohorts when the COVID-19 pandemic hit Norway. Since March 2020, participants have been invited to answer electronic questionnaires every 14 days with questions regarding illness, testing for COVID-19, and more. The response rates to the questionnaires distributed between March 2020 and May 2021 have been 50–80% in both cohorts.

For the current study, 146 516 eligible participants included all adult cohort members who were invited to answer a questionnaire in January 2021 about tobacco use. An inclusion criterion for the main analysis (Sample 1) was available national registry data on SARS-CoV-2 infection status, which was obtained for MoBa only.

**Fig. 1:**
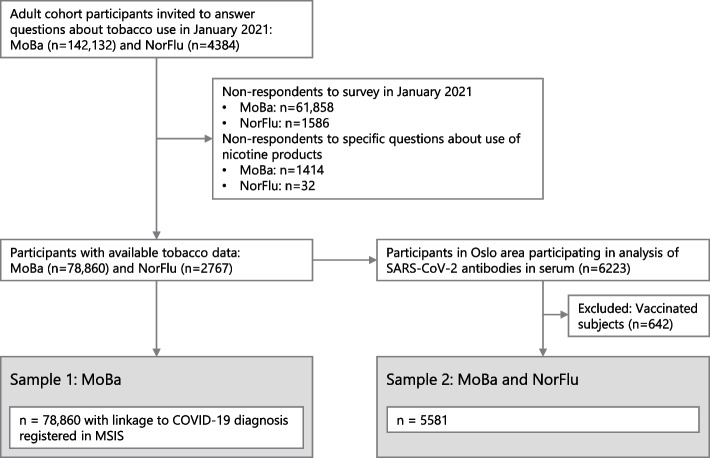
Flowchart for inclusion. MoBa participants in Sample 2 (*n* = 5446) are also part of Sample 1

For analyses using seropositivity as outcome (Sample 2), the inclusion criterion was available data from analysis of SARS-CoV-2 antibodies in serum, which was performed in a subsample from both cohorts. A total of 6917 eligible participants from both cohorts residing in the Oslo area were recruited for antibody analysis. Of these, 6223 (90%) had available information about tobacco use. We excluded MoBa participants who according to SYSVAK data had been vaccinated prior to antibody assessment (*n* = 642), Fig. 1.

### Outcome variables

Our primary outcome was having acquired a SARS-CoV-2 infection, measured as i) a positive test for SARS-CoV-2 based on PCR obtained from The Norwegian Surveillance System for Communicable Diseases (MSIS), registered in the period March 6^th^ 2020 to May 25^th^ 2021 or ii) the presence of antibodies for SARS-CoV-2, collected April 28^th^ 2020 to April 29^th^ 2021. Seropositivity was obtained as a conclusion made by the laboratory at Oslo University Hospital, based on concentrations of antibodies to SARS-CoV-2 the receptor binding domain of the spike protein (RBD) and nucleocapsid protein, measured as previously described [13, 14].

A secondary outcome was whether participants had been tested for SARS-CoV-2 (both negative and positive results). We defined being tested as participants who answered “yes” at least once to the following question: “Have you been tested for coronavirus infection the last 14 days?” from 30 questionnaires administered every 14 days between March 31th 2020 and May 11^th^ 2021. Participants answering “no” and “don’t know” were grouped together.

### Exposure variables

Current cigarette smoking status was obtained from two questionnaires, one was administered in June 2020 and another one in January 2021 (Supplementary Table S1). Participants were grouped as cigarette smokers if they reported daily or occasional smoking in either of the two questionnaires. Snus and other tobacco use (E-cigarette with nicotine, nicotine gum, or “other nicotine products”) were asked in January 2021 only and assumed to represent tobacco use in the entire study period. Participants who answered “yes” to questions about current tobacco use were also asked to report number of cigarettes smoked weekly and/or number of snus boxes per month.

### Other variables

From the existing cohort databases, we included variables on the participants’ age (5- and 10- year intervals for NorFlu/continuous for MoBa, obtained through linkage with MBRN) and gender (defined by cohort member role as mother or father). County of residence was obtained through linkage to The Norwegian Population Registry and grouped into six geographical regions (Oslo and Viken; Vestfold, Telemark, Agder, Rogaland; Vestland; Møre og Romsdal, Innlandet; Trøndelag; Nordland, Troms and Finnmark). As proxies of number of social contacts, we included information on use of home office, job loss, or sick leave (yes/no, reported April and October 2020), as well as number of people in the household. As proxy of socioeconomic status, we included educational level (less than high school, high school, college ≤ 4 years, more than 4 years college) reported by participants in August 2021. For those with missing current information (19%), we used most recent reported education level recorded in the existing cohort database (reported at several occasions from recruitment and up to 2015). We also included information on body mass index (BMI, calculated from height and weight self-reported in January 2021) and underlying chronic illness (asthma, cancer, cardiovascular disease, hypertension, diabetes, other, reported March/April 2020). From each round of 30 questionnaires administered biweekly between March 31th 2020 and May 11^th^ 2021 we also included information about the reason for taking a SARS-CoV-2 test, to separate between those who were tested due to symptoms (defined as those with at least one test due to symptoms), and those who were tested for other reasons (defined as those with at least one test due to work situation, travels, or similar).

### Statistical analysis

We estimated associations between tobacco use and SARS-CoV-2 infection using binomial regression models with logit link function and robust standard errors. All associations were reported as odds ratio (OR) with 95% confidence intervals (CI). Analyses using the large study sample, Sample 1 (*n* = 78,860), were stratified by gender to account for the dependency within families. Adjusted models included the following covariates as fixed effects: Age, education level, number of household members, and work situation. We have based our analyses on a causal framework (of available variables in our data) illustrated by a directed acyclic graph (DAG) in Supplementary Figure S1. Based on the DAG, a minimal adjustment set of confounders are suggested for estimation of the total effect, including gender, age, socioeconomic status, county, number of household members, and work situation. Chronic illness was considered as a potential mediator on a causal pathway between former/current tobacco use and infection status and not included in the regression models. For analysis of the nationwide sample with diagnosis from registry as outcome, we also adjusted for geographical region. As a secondary analysis we estimated associations between tobacco use and having been tested for SARS-CoV-2, stratified by gender and reason for testing (due to symptoms or due to other reasons) and adjusted for age, educational level, region, number of household members, and work situation. In the analysis with presence of antibodies as outcome, we excluded MoBa participants with at least one registered COVID-19 vaccine dose in SYSVAK. Because we did not have this information for NorFlu participants, the analysis was run with and without inclusion of NorFlu participants, with similar findings.

As sensitivity analyses, we analyzed associations between tobacco use and SARS-CoV-2 infection a) in a subset who reported to have been tested at least once during the study period, to account for testing behavior; and b) stratified by BMI (< 25 or ≥ 25 kg/m^2^), to examine a possible effect modification. We also performed the analysis while excluding those who tested positive for SARS-CoV-2 before June (first assessment of smoking status) to account for exposure misclassification of those who may have stopped smoking after getting infected, and while excluding those who reported smoking in January, but not in June (possible exposure misclassification of “new” smokers). None of the two latter approaches changed our findings (data not shown). Lastly, we excluded vaccinated subjects and performed the analysis in a subset who were not yet vaccinated in May 2021. Statistical analyses were performed in R [15], version 4.1.0, using packages *mice* [16], *lmtest* [17], and *sandwich* [18]*.*

### Missing data

We analyzed associations both with complete case analysis and after imputing missing values in covariates. The total proportion of missing values in covariates was highest for work situation (7.3%), and region (6.9%), followed by number of cohabitants (2.7%) and education level (1.0%). We performed multiple imputation with chained equations and 20 imputations*.* The dataset used for imputation included all covariates, as well as COVID-19 diagnosis and testing behavior. Imputed values did not differ substantially from observed values (Supplementary Table S2). Tables 2 and 3 show results from analyses with imputed missing values in covariates, which did not differ substantially from results from complete case analyses (Supplementary Table S3).

## Results

Current snus use was more common among men (26%) than women (9%), while current cigarette smoking was less common than snus both among men (5%) and women (7%) (Table 1). 1.3% reported to use both cigarette smoke and other nicotine products. Only 1.4% of men and 0.7% of women reported using nicotine products other than snus or cigarettes. Compared with cigarette smokers, snus users were younger, had higher education level and lower BMI (Supplementary Table S4). About 2% were registered with a COVID-19 diagnosis and 49% of women and 39% of men reported to have been tested for SARS-CoV-2. Among those who had been tested at least once in the study period, 3.1% were registered with a COVID-19 diagnosis. In the subsample with available antibody analyses, 3.2% of MoBa participants were registered with a COVID-19 diagnosis and 1.9% were seropositive (Supplementary Table S5).

**Table 1:** Study population characteristics, tobacco use and incident cases of COVID-19 between March 2020 and May 2021

	**Study sample (*n* = 78,860)**	
	**Female (*n* = 48,082)**	**Male (*n* = 30,778)**
**Age (years)**		
30–34	287 (0.6%)	38 (0.1%)
35–39	3621 (7.5%)	938 (3%)
40–44	13,004 (27%)	5931 (19.3%)
45–49	18,720 (38.9%)	11,619 (37.8%)
50–54	10,125 (21.1%)	8152 (26.5%)
55–59	2105 (4.4%)	2944 (9.6%)
60 +	154 (0.3%)	1115 (3.6%)
Missing	66 (0.1%)	41 (0.1%)
**Educational level**		
< High school	1015 (2.1%)	942 (3.1%)
High school	10,614 (22.1%)	9212 (29.9%)
College ≤ 4 years	20,189 (42%)	10,307 (33.5%)
College > 4 years	15,825 (32.9%)	9931 (32.3%)
Missing/other	439 (0.9%)	386 (1.3%)
**BMI, kg/m**^**2**^		
< 18.5	514 (1.1%)	42 (0.1%)
18.5–24.9	23,865 (49.6%)	9907 (32.2%)
25–29.9	14,802 (30.8%)	14,992 (48.7%)
30–34.9	5740 (11.9%)	4430 (14.4%)
≥ 35	2312 (4.8%)	1133 (3.7%)
Missing	849 (1.8%)	274 (0.9%)
**Current tobacco use**		
Non-user of tobacco^a^	40,912 (85.1%)	21,689 (70.5%)
Current tobacco user (cigarettes, snus or other)^a^	7170 (14.9%)	9089 (29.5%)
Cigarette smoker^b^	3224 (6.7%)	1550 (5.0%)
User of snus or other nicotine^c^	4518 (9.4%)	8023 (26.1%)
**Registry diagnosis of COVID-19 (MSIS)**		
No	47,127 (98%)	30,174 (98%)
Yes	955 (2%)	604 (2%)
**Have been tested for SARS-CoV-2 by PCR (self-reported)**		
Not tested	23,266 (48.4%)	17,930 (58.3%)
Tested	24,816 (51.6%)	12,848 (41.7%)

^a^User of any type of tobacco, including cigarettes, snus, E-cigarette with nicotine, nicotine gum or other nicotine-containing product. Study sample was restricted to subjects with this information

^b^Any use of cigarette smoke. Missing information for *n* = 79 female and *n* = 122 male subjects

^c^Any use of snus, E-cigarette with nicotine, nicotine gum or other nicotine-containing product. Missing information for *n* = 64 female and *n* = 22 male subjects

We found no associations between current cigarette smoking or snus use and a COVID-19 diagnosis among men (Table 2). For women, the associations between tobacco use and a diagnosis were conflicting, indicating that current cigarette smoking was negatively associated with a diagnosis (adjusted OR 0.51, 95% CI 0.35, 0.75), while no associations were found for snus use (adjusted OR 1.07, 95% CI 0.86, 1.34). The direction of the effect estimate was similar for cigarette smoking when using seropositivity as outcome variable, but with large confidence intervals due to small sample size (Supplementary Table S6). The negative association between cigarette smoking and a diagnosis was only seen in women with BMI ≥ 25 kg/m^2^, and not among women with BMI < 25 kg/m^2^ (Supplementary Table S7). When repeating the analysis in a subset who reported that they had been tested for SARS-CoV-2 at least once during the study period (irrespective of test results), the negative association between cigarette smoking and a diagnosis was similar for women, Supplementary Table S8. No association was found between snus use and a diagnosis in this subset. Exclusion of vaccinated subjects did not change the associations between tobacco use and COVID-19 diagnosis (Supplementary Table S9).

**Table 2:** Associations between tobacco use and a registry diagnosis of COVID-19^a^

		**COVID-19 diagnosis (MSIS)**		
	**Total, N**	**n with diagnosis**	**Unadjusted OR (95% CI)**	**Adjusted**^a^ **OR (95% CI)**
**MEN**				
Non-user of tobacco	21,689	426	1.00 (Ref.)	1.00 (Ref.)
Current tobacco user	9089	178	1.00 (0.84, 1.19)	0.99 (0.83, 1.18)
Cigarettes only^b^	1044	16	0.78 (0.48, 1.28)	0.76 (0.46, 1.26)
Snus or other nicotine only^c^	7417	152	1.04 (0.87, 1.25)	1.03 (0.85, 1.24)
1–3 cans/month	1084	20	0.94 (0.60, 1.46)	0.89 (0.56, 1.40)
4–8 cans/month	2459	40	0.83 (0.60, 1.14)	0.85 (0.61, 1.17)
≥ 9 cans/month	3351	83	1.26 (1.00, 1.59)	1.22 (0.96, 1.54)
**WOMEN**				
Non-user of tobacco	40,912	834	1.00 (Ref.)	1.00 (Ref.)
Current tobacco user	7170	121	0.83 (0.69, 1.00)	0.82 (0.67, 0.99)
Cigarettes only^b^	2588	28	0.53 (0.36, 0.77)	0.51 (0.35, 0.75)
Snus or other nicotine only^c^	3867	85	1.08 (0.86, 1.34)	1.07 (0.86, 1.34)
1–3 cans/month	784	21	1.31 (0.86, 2.01)	1.30 (0.84, 1.99)
4–8 cans/month	1186	27	1.12 (0.76, 1.63)	1.14 (0.78, 1.68)
≥ 9 cans/month	1120	22	0.96 (0.63, 1.47)	0.91 (0.59, 1.40)

^a^ Adjusted for age, education level, region, number of household members, and work situation. Missing values in covariates were handled by multiple imputation

^b^ No snus or other nicotine products

^c^ No cigarettes

When studying testing behavior as outcome, we found inconclusive evidence of an association with current cigarette smoking (OR 0.99, 91% CI 0.87, 1.12 for men and OR 1.15, 95% CI 1.06, 1.25 for women). Both male and female snus users had higher odds for being tested for COVID-19 (OR 1.10, 95% CI 1.04, 1.16 for men and OR 1.22, 95% CI 1.14, 1.30) for women (Table 3). In the sensitivity analysis of subsets who had recorded reasons for testing, we found that associations with testing among smokers were similar for different reasons for testing (tested due to symptoms or not), Supplementary Table S10. The increased OR for testing among male snus users was only significant among those tested for other reasons than presence of symptoms, while no associations were found for those who tested themselves due to symptoms. For female snus users, we found associations with testing both due to symptoms and for other reasons (Supplementary Table S10).

**Table 3:** Associations between cigarette smoking, snus use and testing behavior (subjects who reported to have been tested for COVID-19 at least once in the period March 2020 to March 2021 vs. subjects with no test in the period)

		**Have been tested for COVID-19 in the period March 2020 to March 2021**		
	**Total, N**	**n tested**	**Unadjusted OR (95% CI)**	**Adjusted**^**a**^** OR (95% CI)**
**MEN**				
Non-user of tobacco	21,689	8927	1.00 (Ref.)	1.00 (Ref.)
Current tobacco user	9089	3921	1.08 (1.03, 1.14)	1.09 (1.04, 1.15)
Cigarettes only^b^	1044	406	0.91 (0.80, 1.03)	0.99 (0.87, 1.12)
Snus or other nicotine only^c^	7417	3242	1.11 (1.05, 1.17)	1.10 (1.04, 1.16)
**WOMEN**				
Non-user of tobacco	40,912	20,913	1.00 (Ref.)	1.00 (Ref.)
Current tobacco user	7170	3903	1.14 (1.09, 1.20)	1.18 (1.12, 1.24)
Cigarettes only^b^	2588	1355	1.05 (0.97, 1.14)	1.15 (1.06, 1.25)
Snus or other nicotine only^c^	3867	2180	1.24 (1.16, 1.32)	1.22 (1.14, 1.30)

^a^Adjusted for age, education level, region, number of household members, and work situation. Missing values in covariates were handled by multiple imputation

^b^No snus or other nicotine products

^c^No cigarettes

## Discussion

In this large population-based cohort, we estimated associations between cigarette smoking or other nicotine (mainly snus) use and having had a positive test for SARS-CoV-2 between March 2020 to May 2021. During the study period, the SARS-CoV-2 infection rate in Norway was still low, and only 2% in our study sample had a positive SARS-Cov-2 test. Also, most subjects were still unvaccinated, which enabled investigation of these associations without potential effect modification by vaccination hesitancy and/or effect. We found no associations between nicotine use, with either cigarette smoking or snus use, and a COVID-19 diagnosis among men. Similarly, snus use was not associated with a COVID-19 diagnosis in women. In contrast, we found negative associations between cigarette smoking and a COVID-19 diagnosis among women. Female smokers were more likely to be tested compared to non-smoking women, which could contribute a higher occurrence of positive tests. However, both cigarette smokers and snus users were more likely to take a COVID-19 test than non-users of tobacco, thus the difference in associations with SARS-CoV-2 between these groups were not explained by testing behavior alone.

Hypotheses for both protective and detrimental effects of smoking on COVID-19 have been proposed in the literature. Early epidemiological studies reported an underrepresentation of smokers among patients hospitalized with COVID-19 [19]. However, it has been speculated that epidemiological evidence may be biased due to underdiagnosed COVID-19 among smokers, misreporting of smoking among hospitalized patients, introduction of collider bias due to adjustment strategies, or failure to adjust for former smoking [20, 21]. Also, associations between smoking status and test-positivity (using population controls) in a non-universal testing setting may be affected by residual confounding [22]. Conversely, a detrimental role of nicotine has been related to its known effect on immunomodulation and regulation of angiotensin-converting enzyme 2 (ACE2) receptors, which binds the SARS-CoV-2 virus and facilitates cell entry [23]. It has been shown that ever smokers have higher gene expression of ACE2 receptor in lung tissues compared with never smokers [23], thus suggesting that smokers, either current or former, have increased risk of viral binding and entry into the lungs. This finding is supported by a study from UK, where genetically predicted propensity to start smoking, as well as predicted higher number of cigarettes were associated with increased risk of SARS-CoV-2 infection [24].

In two recent studies based on Finnish and Swedish general population samples, we did not find conclusive evidence of an association between current smoking and COVID-19 diagnosis [25, 26], when compared to non-users of tobacco. In the Swedish study, associations between current smoking and COVID-19 (adjusted RR 0.68, 95% CI 0.61, 0.75) were similar to this study on Norwegian data (ORs 0.76, 95% CI 0.46, 1.26 for men and 0.51, 95% CI 0.35, 0.75 for women) [26]. In the Finnish study, the relative risk of a COVID-19 diagnosis for current smokers was 1.12 (95% CI 0.65–1.94) [25]. For snus use, the estimated risk in the Swedish study was 1.09 (95% CI 0.99, 1.21) among men and 1.15 (95% 1.00, 1.33) among women. The Finnish snus users had increased risk (RR 1.68, 95% CI 1.02–2.75) of COVID-19 when compared to non-users of tobacco. Held together, the findings on snus use in the three Nordic studies do not support the hypothesis of a protective effect of nicotine on SARS-CoV-2 infection. The lack of an association between snus use and COVID-19 diagnosis in the current Norwegian study may suggest that the increased risk of COVID-19 among Finnish and Swedish snus users may be explained by social and/or environmental mechanisms, rather than a biological effect of nicotine.

Cigarette smokers and snus users in our study differed in characteristics and behavior, and potential bias in analyses of the two exposures may also differ, which may contribute to the inconsistency in our findings. For instance, snus users were more likely to have higher education and cigarette smokers lower education, suggesting that these groups may differ in many unmeasured aspects associated with educational level. Also, snus users may have higher occupational risk of acquiring an infection, as snus use may be more acceptable than cigarette smoking in settings where people meet, especially indoor. We found that female snus users had increased odds of having been tested for SARS-CoV-2 than non-tobacco users. This could theoretically hide a decreased risk of infection among snus users in our analysis if the prevalence of COVID-19 in the control group (non-tobacco users) was underestimated. However, when restricting our analyses to only those who had been tested, we still found no association between snus use and a COVID-19 diagnosis.

There are several limitations of this study. We did not have recent information about former tobacco use. Use of snus and other nicotine-containing products was reported during the second wave of increased infection rates in Norway, and for some participants after acquiring an infection. This may have resulted in exposure misclassification of those who changed their snus use, for instance after infection. Despite the large sample size, the SARS-CoV-2 infection rates in Norway during the pre-vaccination period were low, which reduced the statistical power and increased the uncertainty in our analyses. Due to this limitation, we did not investigate how different virus variants, season, or other time-varying factors during the study period may have affected the associations. While positive tests were obtained from a registry based on PCR tests, we used self-reported data on testing behavior. Some participants may have reported other types of testing (i.e. rapid antigen test) that was not performed through the health services. Another draw-back with self-reported data is that those who experienced symptoms of COVID-19 may be more likely to contribute data in the ongoing surveys than those who were asymptomatic. Yet, the proportion of participants diagnosed with COVID-19 in this study reflects the (low) infection rates in Norway [27]. The prevalence of cigarette smoking in this study was lower than in the general population, while the proportion of snus users was comparable [7]. The underrepresentation of cigarette smokers and higher socio-economic status were introduced at the initial recruitment to MoBa [28]. The cohorts included in this study were recruited among pregnant women and their partners during 1999–2008 (MoBa) and 2009–2010 (NorFlu) and our study sample only include adults who have children. We do not know whether the associations reported are different in adults without children or in adults above 65 years of age.

A main strength of our study is that we could examine both registry-based diagnosis, seroprevalence results and testing behavior as outcomes, with two commonly used nicotine products (cigarettes and snus) as exposures. We were also able to compare testing behavior between users and non-users of nicotine products. Another main strength is the large sample size and high participation rate. Both genders and participants from all parts of the country are well represented, and there is a wide age span. Using pre-existing cohorts as opposed to panels recruited during the pandemic may be advantageous because they are less subject to selection bias.

In conclusion, our study showed inconsistent associations between nicotine use and being infected with SARS-CoV-2 in Norway during the pre- and early vaccination periods. Cigarette smoking was negatively associated with acquiring a SARS-CoV-2 infection among women only, while no associations were found among men or women between snus use and a COVID-19 diagnosis. Testing behavior in nicotine users (smokers and snus users) differed from non-users, but were similar for snus users and smokers. The absence of an association between snus use and a SARS-CoV-2 infection in a population with prevalent snus use does not support the hypothesis of a protective effect of nicotine.

## Supplementary Information

**Additional file 1.** **Supplementary Table S1.** Frequency of cigarette smoking in MoBa (*n*=78,860), comparing answers at the two timepoints. **Supplementary Table S2.** Results from multiple imputation with chained equations of missing values in covariates. Total sample size is *n*=78,860. **Supplementary Table S3.** Complete case analysis of associations between tobacco use and a COVID-19 or testing behavior. Unadjusted and adjusted analyses were performed on the same dataset, excluding cases with missing observations in covariates. **Supplementary Table S4.** Study population characteristics and incident cases of COVID-19 between March 2020 and May 2021, among exclusive cigarette smokers, exclusive snus users and non-users of tobacco. **Supplementary Table S5.** Study population characteristics in the two cohorts with available SARS-CoV-2 antibody data. **Supplementary Table S6.** Associations between tobacco use and SARS-CoV-2 seropositivity. **Supplementary Table S7.** Tobacco use and MSIS diagnosis, in gender and BMI strata, complete case analysis. **Supplementary Table S8.** Tobacco use and MSIS diagnosis in a subset who had been tested at least once during the study period, complete case analysis. **Supplementary Table S9.** Tobacco use and MSIS diagnosis in a subset who were unvaccinated by May 2021, complete case analysis. **Supplementary Table S10.** Associations between tobacco use and having been tested for SARS-CoV-2, stratified by gender and having been tested because of symptoms or not. **Figure S1.** Suggested causal framework for the association between current smoking/tobacco use and risk of being infected with SARS-CoV-2. Minimal adjustment set of confounders for estimation of total effect includes sex, age, SES, county, number of household members, and work situation (home office/lost job). Chronic illness was considered as a potential mediator on a causal pathway between former/current tobacco use and infection status and not included in adjusted models

## Data Availability

Data from the cohorts used in this study are managed by the national health register holders in Norway (Norwegian Institute of Public Health) and can be made available to researchers, provided approval from the Regional Committees for Medical and Health Research Ethics (REC), compliance with the EU General Data Protection Regulation (GDPR) and approval from the data owners. The consent given by the participants does not open for storage of data on an individual level in repositories or journals. Researchers who want access to data sets for replication should apply through helsedata.no. Access to data sets requires approval from The Regional Committee for Medical and Health Research Ethics in Norway and an agreement with MoBa.
