# Supplementary material for: Current tobacco use and SARS-CoV-2 infection in two Norwegian population-based cohorts
Source: BMC Public Health. 2023 May 10;23:846. doi: 10.1186/s12889-023-15822-5 (PMC10170041; doi:10.1186/s12889-023-15822-5)
Supplement: Supplementary file 1 — Additional file 1. Supplementary Table S1. Frequency of cigarette smoking in MoBa (n=78,860), comparing answers at the two timepoints. Supplementary Table S2. Results from multiple imputation with chained equations of missing values in covariates. Total sample size is n=78,860. Supplementary Table S3. Complete case analysis of associations between tobacco use and a COVID-19 or testing behavior. Unadjusted and adjusted analyses were performed on the same dataset, excluding cases with missing observations in covariates. Supplementary Table S4. Study population characteristics and incident cases of COVID-19 between March 2020 and May 2021, among exclusive cigarette smokers, exclusive snus users and non-users of tobacco. Supplementary Table S5. Study population characteristics in the two cohorts with available SARS-CoV-2 antibody data. Supplementary Table S6. Associations between tobacco use and SARS-CoV-2 seropositivity. Supplementary Table S7. Tobacco use and MSIS diagnosis, in gender and BMI strata, complete case analysis. Supplementary Table S8. Tobacco use and MSIS diagnosis in a subset who had been tested at least once during the study period, complete case analysis. Supplementary Table S9. Tobacco use and MSIS diagnosis in a subset who were unvaccinated by May 2021, complete case analysis. Supplementary Table S10. Associations between tobacco use and having been tested for SARS-CoV-2, stratified by gender and having been tested because of symptoms or not. Figure S1. Suggested causal framework for the association between current smoking/tobacco use and risk of being infected with SARS-CoV-2. Minimal adjustment set of confounders for estimation of total effect includes sex, age, SES, county, number of household members, and work situation (home office/lost job). Chronic illness was considered as a potential mediator on a causal pathway between former/current tobacco use and infection status and not included in adjusted models [file 12889_2023_15822_MOESM1_ESM.pdf]

SUPPLEMENTARY INFORMATION

Current tobacco use and SARS-CoV-2 infection in two Norwegian population-based cohorts

Ida Henriette Caspersen, Lill Trogstad, Maria Rosaria Galanti, Sakari Karvonen, Sebastián Peña, Ahmed Nabil Shaaban, Siri E. Håberg, Per Magnus

Supplementary Table S1. Frequency of cigarette smoking in MoBa 2

Supplementary Table S2. Results from multiple imputation with chained equations of missing values in covariates. 3

Supplementary Table S3. Complete case analysis of associations between tobacco use and a COVID-19 or testing behavior 4

Supplementary Table S4. Study population characteristics and incident cases of COVID-19 between March 2020 and May 2021, among exclusive cigarette smokers, exclusive snus users and non-users of tobacco. 5

Supplementary Table S5. Study population characteristics in the two cohorts with available SARS-CoV-2 antibody data. 6

Supplementary Table S6. Associations between tobacco use and SARS-CoV-2 seropositivity. 7

Supplementary Table S7. Tobacco use and MSIS diagnosis, in gender and BMI strata, complete case analysis... 8

Supplementary Table S8. Tobacco use and MSIS diagnosis in a subset who had been tested at least once during the study period, complete case analysis. 9

Supplementary Table S9. Associations between tobacco use and having been tested for SARS-CoV-2, stratified by gender and having been tested because of symptoms or not. 10

Figure S1. Suggested causal framework for the association between current smoking/tobacco use and risk of being infected with SARS-CoV-2. 12

Supplementary Table S1. Frequency of cigarette smoking in MoBa (n=78,860), comparing answers at the two timepoints.

	Cigarette smoking, January 2021			
**Cigarette smoking, June 2020**	No	Occasional	Daily	Missing
No	63826	462	111	48
Occasional	979	1485	248	0
Daily	292	300	2741	0
Missing	8167	107	92	2

Supplementary Table S2. Results from multiple imputation with chained equations of missing values in covariates. Total sample size is n=78,860.

	n (%) with missing values	Observed values	Imputed values
**Age (years), mean**	107 (0.1)	47.2	47.2
**Educational level, %**	825 (1.0)		
< High school		25.1	22.0
High school		25.4	24.1
College ≤4 years		39.1	40.2
College >4 years		33.0	33.5
**Work situation, %**	5789 (7.3)		
No change		84.6	84.9
Home-based office		13.3	13.2
Lost job, temporary layoff or sick leave		2.0	2.0
**Number of cohabitants, %**	2105 (2.7)		
0		1.0	1.0
1		4.3	4.4
2		15.7	15.7
3		44.4	44.1
4		26.7	26.9
5 or more		8.0	8.0
**Region, %**	5432 (6.9)		
Oslo, Viken		39.0	38.9
Rogaland, Agder, Vestfold, Telemark		19.7	19.8
Vestland		14.9	15.0
Innlandet, Møre and Romsdal		12.4	12.2
Trøndelag		8.4	8.4
Nordland, Troms and Finnmark		5.6	5.7

Supplementary Table S3. Complete case analysis of associations between tobacco use and a COVID-19 or testing behavior. Unadjusted and adjusted analyses were performed on the same dataset, excluding cases with missing observations in covariates.

		COVID-19 diagnosis (MSIS)			Have been tested for COVID-19		
	**Total, N**	**n with diagnosis**	**Unadjusted OR (95% CI)**	**Adjusted [a] OR (95% CI)**	**n**	**Unadjusted [a] OR (95% CI)**	**Adjusted [b] OR (95% CI)**
**MEN**							
Non-user of tobacco	18495	360	1.00 (Ref.)	1.00 (Ref.)	7761	1.00 (Ref.)	1.00 (Ref.)
Current tobacco user	7663	150	1.01 (0.83, 1.21)	0.99 (0.82, 1.20)	3354	1.08 (1.02, 1.14)	1.08 (1.02, 1.14)
Cigarettes only [c]	896	14	0.81 (0.48, 1.38)	0.82 (0.49, 1.37)	345	0.86 (0.75, 0.99)	0.97 (0.84, 1.11)
Snus or other nicotine only [d]	5854	116	1.03 (0.84, 1.27)	1.02 (0.84, 1.25)	2615	1.11 (1.04, 1.18)	1.10 (1.03, 1.16)
1-4 cans/month	831	11	0.69 (0.38, 1.25)	0.67 (0.38, 1.19)			
5-9 cans/month	1935	33	0.89 (0.62, 1.27)	0.91 (0.65, 1.28)			
10-14 cans/month	2643	64	1.26 (0.97, 1.64)	1.22 (0.94, 1.57)			
**WOMEN**							
Non-user of tobacco	34236	643	1.00 (Ref.)	1.00 (Ref.)	17859	1.00 (Ref.)	1.00 (Ref.)
Current tobacco user	5975	100	0.89 (0.72, 1.10)	0.88 (0.71, 1.08)	3309	1.14 (1.08, 1.2)	1.17 (1.10, 1.23)
Cigarettes only [c]	2241	26	0.62 (0.42, 0.91)	0.56 (0.38, 0.83)	1169	1.00 (0.92, 1.09)	1.08 (0.99, 1.18)
Snus or other nicotine only [d]	3148	67	1.13 (0.88, 1.45)	1.14 (0.90, 1.45)	1823	1.26 (1.17, 1.36)	1.23 (1.14, 1.32)
1-4 cans/month	609	17	1.49 (0.92, 2.39)	1.42 (0.90, 2.26)			
5-9 cans/month	950	23	1.29 (0.85, 1.94)	1.29 (0.86, 1.93)			
10+ cans/month	917	16	0.93 (0.57, 1.52)	0.93 (0.57, 1.49)			

[a] The same dataset is used for both unadjusted and adjusted analysis.
[b] Adjusted for age, education, region, number of household members, work situation.
[c] No snus or other nicotine products
[d] No cigarettes

Supplementary Table S4. Study population characteristics and incident cases of COVID-19 between March 2020 and May 2021, among exclusive cigarette smokers, exclusive snus users and non-users of tobacco.

	WOMEN			MEN		
**Age (years)**	**Exclusive cigarette smokers**	**Exclusive snus users**	**No tobacco**	**Exclusive cigarette smokers**	**Exclusive snus users**	**No tobacco**
30-34	29 (1.1%)	102 (3.2%)	310 (0.8%)	6 (0.6%)	30 (0.4%)	31 (0.1%)
35-39	249 (9.6%)	601 (18.7%)	3585 (8.8%)	40 (3.8%)	400 (5.7%)	825 (3.8%)
40-44	731 (28.2%)	1120 (34.8%)	11911 (29.1%)	158 (15.1%)	1834 (26.1%)	4561 (21%)
45-49	917 (35.4%)	932 (29%)	15409 (37.7%)	374 (35.8%)	2628 (37.5%)	7844 (36.2%)
50-54	530 (20.5%)	336 (10.4%)	7361 (18%)	288 (27.6%)	1449 (20.6%)	5148 (23.7%)
55-59	110 (4.3%)	36 (1.1%)	1328 (3.2%)	117 (11.2%)	365 (5.2%)	1844 (8.5%)
60+	9 (0.3%)	1 (0%)	78 (0.2%)	56 (5.4%)	76 (1.1%)	711 (3.3%)
Missing	13 (0.5%)	89 (2.8%)	930 (2.3%)	5 (0.5%)	235 (3.3%)	725 (3.3%)
**Educational level**						
< High school	150 (5.8%)	118 (3.7%)	672 (1.6%)	72 (6.9%)	253 (3.6%)	538 (2.5%)
High school	1002 (38.7%)	931 (28.9%)	8185 (20%)	469 (44.9%)	2410 (34.3%)	5903 (27.2%)
College ≤4 years	914 (35.3%)	1304 (40.5%)	17463 (42.7%)	315 (30.2%)	2413 (34.4%)	7290 (33.6%)
College >4 years	494 (19.1%)	812 (25.2%)	14251 (34.8%)	176 (16.9%)	1831 (26.1%)	7713 (35.6%)
Missing/other	28 (1.1%)	52 (1.6%)	341 (0.8%)	12 (1.1%)	110 (1.6%)	245 (1.1%)
**BMI, kg/m^2**						
<18.5	28 (1.1%)	59 (1.8%)	412 (1%)	6 (0.6%)	8 (0.1%)	25 (0.1%)
18.5-24.9	1042 (40.3%)	1678 (52.2%)	20506 (50.1%)	309 (29.6%)	2156 (30.7%)	7174 (33.1%)
25-29.9	919 (35.5%)	972 (30.2%)	12443 (30.4%)	505 (48.4%)	3467 (49.4%)	10491 (48.4%)
30-34.9	417 (16.1%)	349 (10.8%)	4811 (11.8%)	173 (16.6%)	1064 (15.2%)	3026 (14%)
≥35	145 (5.6%)	124 (3.9%)	1982 (4.8%)	45 (4.3%)	272 (3.9%)	763 (3.5%)
Missing	37 (1.4%)	35 (1.1%)	758 (1.9%)	6 (0.6%)	50 (0.7%)	210 (1%)
**Registry diagnosis of COVID-19 (MSIS)**						
No	2560 (98.9%)	3145 (97.8%)	40078 (98%)	1028 (98.5%)	6875 (98%)	21263 (98%)
Yes	28 (1.1%)	72 (2.2%)	834 (2%)	16 (1.5%)	142 (2%)	426 (2%)
**Have been tested for SARS-CoV-2 by PCR (self-reported)**						
Not tested	1233 (47.6%)	1396 (43.4%)	19999 (48.9%)	638 (61.1%)	3950 (56.3%)	12762 (58.8%)
Tested	1355 (52.4%)	1821 (56.6%)	20913 (51.1%)	406 (38.9%)	3067 (43.7%)	8927 (41.2%)

Supplementary Table S5. Study population characteristics in the two cohorts with available SARS-CoV-2 antibody data.

	MoBa subsample with antibody data (n=5446), n (%)	NorFlu subsample with available antibody data (n=135), n (%)
**Women, n (%)**	3091 (57)	135 (100)
**Age (years)**		
25-34	9 (0.2)	3 (2.2)
35-39	239 (4.4)	16 (11.9)
40-44	1371 (25.2)	64 (47.4)
45-49	2150 (39.5)	45 (33.3)
50-54	1178 (21.6)	7 (5.2)
55-59	318 (5.8)	0 (0)
60+	83 (1.5)	0 (0)
**Educational level**		
< High school	50 (0.9)	3 (2.2)
High school	735 (13.5)	10 (7.4)
College ≤4 years	1918 (35.2)	42 (31.1)
College >4 years	2713 (49.8)	60 (44.4)
Missing/other	30 (0.6)	20 (14.8)
**BMI, kg/m^2**		
<18.5	49 (0.9)	3 (2.2)
18.5-24.9	2811 (51.6)	90 (66.7)
25-29.9	1886 (34.6)	32 (23.7)
30-34.9	478 (8.8)	9 (6.7)
≥35	161 (3)	1 (0.7)
Missing	61 (1.1)	0 (0)
**Current tobacco use**		
Non-user of tobacco	4395 (80.7)	114 (84.4)
Current tobacco user (smoking, snus or other)	1051 (19.3)	21 (15.6)
Cigarette smoker	268 (4.9)	8 (5.9)
User of snus or other nicotine product	6 (0.1)	0 (0)
**SARS-CoV-2 antibodies**		
No	5342 (98)	123 (91.1)
Yes	104 (1.9)	12 (8.9)

Supplementary Table S6. Associations between tobacco use and SARS-CoV-2 seropositivity.

		Seropositive for SARS-CoV-2		
	**Total, n**	**n**	**Unadjusted OR (95% CI)**	**Adjusted OR (95% CI)**
**Imputed covariate data[b] (n=5581)**				
Non-user of tobacco	4509	89	1.00 (Ref.)	1.00 (Ref.)
Current tobacco (smoking, snus or other)	1072	27	1.28 (0.83, 1.98)	1.23 (0.79, 1.93)
Cigarettes only (no snus/other nicotine)	222	4	0.91 (0.33, 2.50)	0.85 (0.30, 2.38)
Snus or other nicotine only (no cigarettes)	790	23	1.49 (0.94, 2.37)	1.40 (0.89, 2.28)
**Complete case analysis (n=5261)**				
Non-user of tobacco	4260	79	1.00 (Ref.)	1.00 (Ref.)
Current tobacco (smoking, snus or other)	1001	25	1.28 (0.83, 1.98)	1.31 (0.82, 2.09)
Cigarettes only (no snus/other nicotine)	197	4	0.91 (0.33, 2.50)	0.96 (0.34, 2.70)
Snus or other nicotine only (no cigarettes)	683	18	1.49 (0.94, 2.37)	1.52 (0.93, 2.48)

[a] Adjusted for age, education level, number of household members, and work situation.
[b] Imputed values from multiple imputation with chained equations. The proportion of missing observations in covariates were: Education (0.9%), number of household members (n=1.8%), and work situation (3.6%).

Supplementary Table S7. Tobacco use and MSIS diagnosis, in gender and BMI strata, complete case analysis.

		COVID-19 diagnosis (MSIS)		
	**Total, N**	**n with diagnosis**	**Unadjusted [a] OR (95% CI)**	**Adjusted [b] OR (95% CI)**
**MEN, BMI <25 kg/m²**				
Non-user of tobacco	5865	99	1.00 (Ref.)	1.00 (Ref.)
Current tobacco user	2197	44	1.19 (0.83, 1.70)	1.23 (0.86, 1.77)
Cigarettes only [b]	266	6	1.34 (0.58, 3.09)	1.40 (0.61, 3.21)
Snus or other nicotine only [c]	1812	37	1.21 (0.83, 1.78)	1.24 (0.85, 1.82)
**MEN, BMI ≥25 kg/m²**				
Non-user of tobacco	11329	229	1.00 (Ref.)	1.00 (Ref.)
Current tobacco user	4961	95	0.95 (0.74, 1.20)	0.93 (0.73, 1.18)
Cigarettes only [b]	625	8	0.63 (0.31, 1.28)	0.62 (0.30, 1.28)
Snus or other nicotine only [c]	4005	79	0.98 (0.75, 1.26)	0.95 (0.73, 1.24)
**WOMEN, BMI <25 kg/m²**				
Non-user of tobacco	17464	331	1.00 (Ref.)	1.00 (Ref.)
Current tobacco user	2845	52	0.96 (0.72, 1.29)	0.98 (0.73, 1.32)
Cigarettes only [b]	919	16	0.92 (0.55, 1.52)	0.91 (0.55, 1.53)
Snus or other nicotine only [c]	1682	35	1.10 (0.77, 1.56)	1.13 (0.79, 1.62)
**WOMEN, BMI ≥25 kg/m²**				
Non-user of tobacco	16181	306	1.00 (Ref.)	1.00 (Ref.)
Current tobacco user	3064	47	0.81 (0.59, 1.10)	0.81 (0.59, 1.11)
Cigarettes only [b]	1294	10	0.40 (0.21, 0.76)	0.38 (0.20, 0.73)
Snus or other nicotine only [c]	1439	31	1.14 (0.79, 1.66)	1.17 (0.80, 1.71)

[a] The same dataset is used for both unadjusted and adjusted analysis.
[b] Adjusted for age, education, region, number of household members, work situation.
[c] No snus or other nicotine products
[d] No cigarettes

Supplementary Table S8. Tobacco use and MSIS diagnosis in a subset who had been tested at least once during the study period, complete case analysis.

		COVID-19 diagnosis (MSIS)		
	**Total, N**	**n with diagnosis**	**Unadjusted [a] OR (95% CI)**	**Adjusted [b] OR (95% CI)**
**MEN**				
Non-user of tobacco	7761	242	1.00 (Ref.)	1.00 (Ref.)
Current tobacco user	3354	112	1.02 (0.83, 1.26)	1.06 (0.84, 1.33)
Cigarettes only [b]	345	12	1.05 (0.61, 1.81)	1.11 (0.63, 1.97)
Snus or other nicotine only [c]	2615	82	1.03 (0.83, 1.29)	1.04 (0.81, 1.33)
**WOMEN**				
Non-user of tobacco	17859	502	1.00 (Ref.)	1.00 (Ref.)
Current tobacco user	3309	75	0.75 (0.60, 0.93)	0.76 (0.60, 0.97)
Cigarettes only [b]	1169	20	0.52 (0.34, 0.79)	0.52 (0.33, 0.83)
Snus or other nicotine only [c]	1823	51	0.94 (0.73, 1.23)	0.97 (0.73, 1.29)

[a] The same dataset is used for both unadjusted and adjusted analysis.
[b] Adjusted for age, education, region, number of household members, work situation.
[c] No snus or other nicotine products
[d] No cigarettes

Supplementary Table S9. Tobacco use and MSIS diagnosis in a subset who were unvaccinated by May 2021, complete case analysis.

		COVID-19 diagnosis (MSIS)		
	**Total, N**	**n with diagnosis**	**Unadjusted [a] OR (95% CI)**	**Adjusted [b] OR (95% CI)**
**MEN**				
Non-user of tobacco	12411	281	1.00 (Ref.)	1.00 (Ref.)
Current tobacco user	5228	116	0.97 (0.80, 1.18)	0.98 (0.78, 1.22)
Cigarettes only [b]	619	11	0.77 (0.44, 1.34)	0.83 (0.46, 1.50)
Snus or other nicotine only [c]	3983	87	1.00 (0.81, 1.23)	0.98 (0.77, 1.24)
**WOMEN**				
Non-user of tobacco	20371	469	1.00 (Ref.)	1.00 (Ref.)
Current tobacco user	3477	72	0.81 (0.65, 1.02)	0.90 (0.69, 1.14)
Cigarettes only [b]	1359	19	0.51 (0.33, 0.79)	0.55 (0.34, 0.87)
Snus or other nicotine only [c]	1783	47	1.06 (0.81, 1.37)	1.16 (0.86, 1.56)

[a] The same dataset is used for both unadjusted and adjusted analysis.
[b] Adjusted for age, education, region, number of household members, work situation.
[c] No snus or other nicotine products
[d] No cigarettes

Supplementary Table S10. Associations between tobacco use and having been tested for SARS-CoV-2, stratified by gender and having been tested because of symptoms or not.

		Have reported to have been tested for SARS-CoV-2 (vs. not tested)		
	**Total, N**	**n tested**	**Unadjusted [a] OR (95% CI)**	**Adjusted [b] OR (95% CI)**
**MEN, tested due to symptoms**				
Non-user of tobacco	15436	4702	1.00 (Ref.)	1.00 (Ref.)
Current tobacco user	6293	1984	1.05 (0.99, 1.12)	1.06 (0.99, 1.13)
Cigarettes only [c]	750	199	0.81 (0.69, 0.96)	0.96 (0.81, 1.13)
Snus or other nicotine only [d]	4796	1557	1.08 (1.01, 1.16)	1.06 (0.99, 1.14)
**MEN, tested for other reasons**				
Non-user of tobacco	13793	3059	1.00 (Ref.)	1.00 (Ref.)
Current tobacco user	5679	1370	1.12 (1.04, 1.20)	1.13 (1.05, 1.21)
Cigarettes only [c]	697	146	0.93 (0.77, 1.12)	0.98 (0.82, 1.18)
Snus or other nicotine only [d]	4297	1058	1.14 (1.06, 1.24)	1.15 (1.06, 1.25)
**WOMEN, tested due to symptoms**				
Non-user of tobacco	29409	13032	1.00 (Ref.)	1.00 (Ref.)
Current tobacco user	5095	2429	1.14 (1.08, 1.22)	1.18 (1.11, 1.25)
Cigarettes only [c]	1906	834	0.98 (0.89, 1.07)	1.09 (0.99, 1.19)
Snus or other nicotine only [d]	2686	1361	1.29 (1.19, 1.40)	1.24 (1.14, 1.34)
**WOMEN, tested for other reasons**				
Non-user of tobacco	21204	4827	1.00 (Ref.)	1.00 (Ref.)
Current tobacco user	3546	880	1.12 (1.03, 1.22)	1.15 (1.06, 1.25)
Cigarettes only [c]	1407	335	1.06 (0.93, 1.20)	1.07 (0.94, 1.22)
Snus or other nicotine only [d]	1787	462	1.18 (1.06, 1.32)	1.22 (1.09, 1.36)

[a] The same dataset is used for both unadjusted and adjusted analysis.
[b] Adjusted for age, education, region, number of household members, work situation.
[c] No snus or other nicotine products
[d] No cigarettes

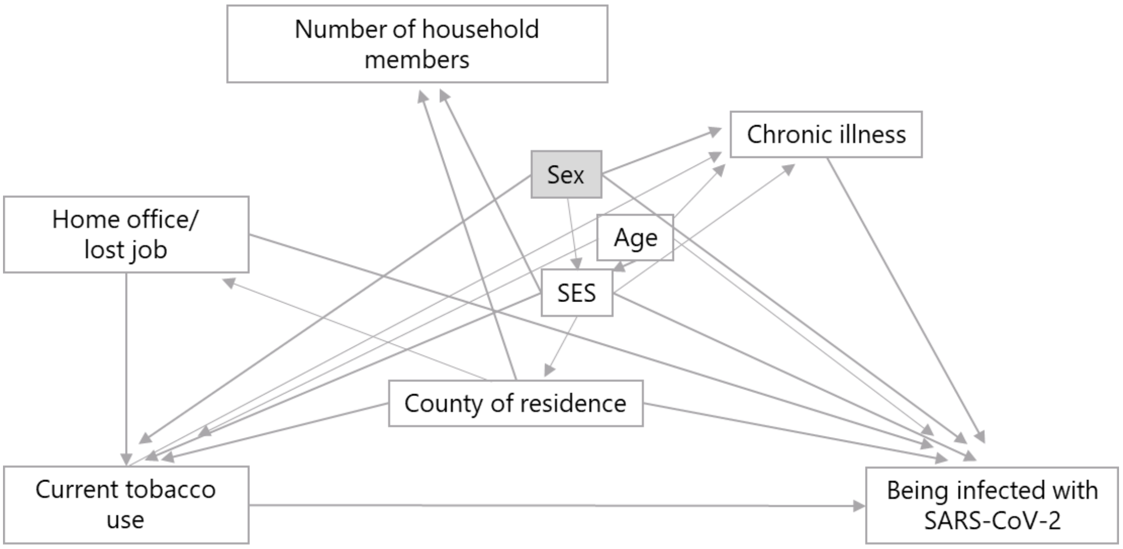

Figure S1. Suggested causal framework for the association between current smoking/tobacco use and risk of being infected with SARS-CoV-2. Minimal adjustment set of confounders for estimation of total effect includes sex, age, SES, county, number of household members, and work situation (home office/lost job). Chronic illness was considered as a potential mediator on a causal pathway between former/current tobacco use and infection status and not included in adjusted models.
